# Phylogenetic identification of *Aeromonas crassostreae* from spinach, first isolate since species description

**DOI:** 10.1016/j.nmni.2026.101731

**Published:** 2026-02-24

**Authors:** Antonio Martínez-Murcia, Adrián García-Sirera

**Affiliations:** a Area of Microbiology, University Miguel Hernández, Orihuela, 03312, Alicante, Spain; b genetic PCR solutions®, Orihuela, 03300, Alicante, Spain

**Keywords:** *Aeromonas crassostreae*, MLPA, ANI, is-DDH, Food

Dear Editor,

The genus *Aeromonas* comprises a group of Gram-negative bacteria widely distributed in aquatic environments, with many species able to cause disease in animals (particularly aquatic) and humans, but also isolated from other environments, including food products. Due to their broad ecological adaptability, these bacteria represent a masterful model within the One Health concept [1]. The taxonomy of this genus is complex, and the 36 species currently described [2] are not always properly identified by phenotypic traits. Multi-locus phylogenetic analysis (MLPA), which compares concatenated nucleotide sequences of a few housekeeping genes, has proved to be highly effective for resolving *Aeromonas* species under a phylogenetic hypothesis [3]. Cutting-edge sequencing technology allows today *in silico* genome comparisons to determine average nucleotide identity (ANI) and *in silico* DNA–DNA hybridization (is-DDH) with respect to reference genomes (i.e., type strains).

A recent study has examined the occurrence of *Aeromonas* species in retail foods, including raw meats and leafy greens, foods relevant to foodborne disease, but less represented in usual studies surveying *Aeromonas* in food products [4]. The study included a MLPA of 31 isolates based on the concatenated sequences of *gyrB*, *rpoD*, *gyrA*, *recA*, *dnaJ*, and *dnaX*, the same genes used on the reference method [3]. The Similar Genome Finder Service on the BV-BRC platform was used to estimate genome distance to the closest species match through Mash/MinHash, and the Type (Strain) Genome Server (https://tygs.dsmz.de/), was used to identify the closest type strain. These genomes were then compared using ANI analysis. The authors identified *A. media*, *A. rivipollensis*, *A. salmonicida*, *A. veronii*, but isolates RHC0020 and RHC0041 were considered unknown *Aeromonas* species. Although Mash/MinHash identified genomes of *A. rivipollensis* and *A. bivalvium* as the closest to these isolates, respectively, the Type (Strain) Genome Server analysis suggested that they correspond to previously undescribed species. This was supported by ANI values below the 95 % cutoff threshold to assign them to any of the species tested, together with MLPA results. However, the analyses only included type strains from four *Aeromonas* species [4].

In our laboratory, the phylogenetic affiliation of these two isolates, RHC0020 and RHC0041, was investigated. We performed a MLPA using a reference tree that includes type and other reference strains when available, for all *Aeromonas* species described to date [2]. The MLPA tree obtained (Fig. 1) clearly showed a phylogenetic assignment of strain RHC0041 to the previously described species *A. crassostreae*. On the other hand, strain RHC0020 clustered in a tight cluster of strains belonging to the so-called *Aeromonas* sp. genomospecies “paramedia” (Clade C), a term introduced by Talagrand-Reboul et al., [5] for a non-yet fully described species of *Aeromonas* inside *A. media* species complex. By using Skani (https://github.com/bluenote-1577/skani), we found that strain RHC0041 showed a maximum ANI value of 98.18 % with *A. crassostreae* strain 25-AL00131, and 94.36 % with *A. bivalvium* CECT 7113, consistent with the previously reported value of 93.72 % for *A. bivalvium* strain NB23SCDHY002 [4]. In our analysis, strain RHC0020 showed an ANI value of 97.86 % with *Aeromonas* sp. strain 3925 and 95.20 % with *Aeromonas* sp. strain Colony414, both belonging to the *Aeromonas* sp. genomospecies “paramedia”. In addition, strain RHC0020 showed 94.28 % ANI with closest species *A. media* CECT 4232^T^ and 94.74 % with *A. rivipollensis* strain P2G1, in agreement with values (93.77-93.99 % and 94.43-94.74 %, respectively) previously reported [4]. Finally, GGDC (Genome-to-Genome Distance Calculator 3.0; https://ggdc.dsmz.de/) was employed to estimate is-DDH and genome of RHC0041 yielded a value of 81.90 % when compared with that of *A. crassostreae* strain 25-AL00131. However, is-DDH was only 54.20 % when compared to *A*. *bivalvium* CECT 7113. On the other hand, strain RHC0020 showed is-DDH values of 81.00 % and 64.00 % to *Aeromonas* sp. genomospecies “paramedia” strains 3925 and Colony414, respectively, but only 54.40 % with type strain CECT 4232^T^ of *A. media* and 57.30 % with *A. rivipollensis* strain P2G1.

**Fig. 1:**
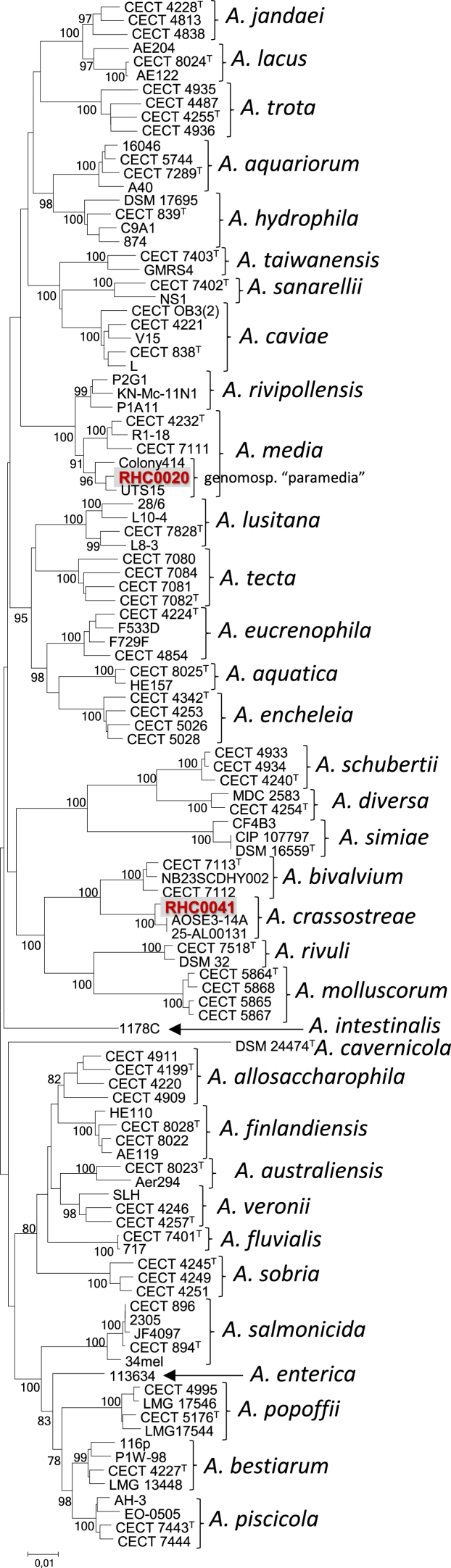
Multilocus phylogenetic analysis (MLPA) obtained from the concatenated sequences of housekeeping genes *gyrB*, *rpoD*, and *recA* (N-J method; total of 1708 bp) showing the relationships of all known *Aeromonas* species and isolates RHC0020 and RHC0041. Numbers at nodes indicate bootstrap values (percentage of 1000 replicates). Bar, 0.01 estimated nucleotide substitutions per site. All sequence data were downloaded from the NCBI Website.

Consequently, we concluded that strain RHC0020 is part of the *Aeromonas* sp. genomospecies “paramedia” within the *A. media* complex and, also, that strain RHC0041, rather than an undescribed species, belongs to *A. crassostreae*, a species originally described with a single isolate. The genome of *A. crassotreae* strain 25-AL00131 was deposited in the NCBI by November 2025 and its isolation source is unknown. This strain and RHC0041, isolated earlier, represent the first additional isolated strains reported since the original species description in 2016. Overall, identification by MLPA requires a comprehensive set of reference data to be include in the analysis.

## CRediT authorship contribution statement

**Antonio Martínez-Murcia:** Conceptualization, Formal analysis, Writing – original draft, Writing – review & editing. **Adrián García-Sirera:** Conceptualization, Formal analysis, Writing – original draft, Writing – review & editing.

## Ethical approval

Not applicable.

## Funding

No funding has been received for this work.

## Declaration of competing interest

The authors declare that they have no known competing financial interests or personal relationships that could have appeared to influence the work reported in this paper.
